# Gestational Diabetes Mellitus: The Impact of Carbohydrate Quality in Diet

**DOI:** 10.3390/nu11071549

**Published:** 2019-07-09

**Authors:** Tiziana Filardi, Francesca Panimolle, Clara Crescioli, Andrea Lenzi, Susanna Morano

**Affiliations:** 1 Department of Experimental Medicine, “Sapienza” University, Viale del Policlinico 155, 00161 Rome, Italy; 2 Department of Movement, Human and Health Sciences, Section of Health Sciences, Unit of Endocrinology, Università degli Studi di Roma “Foro Italico”, 00135 Rome, Italy

**Keywords:** gestational diabetes mellitus, GDM, low glycaemic index, low glycaemic load, low glycaemic index diet, low-GI diet, LGI diet, incidence of GDM, GDM outcomes

## Abstract

Gestational diabetes mellitus (GDM) is defined as “glucose intolerance that is first diagnosed during pregnancy”. Mothers with GDM and their infants may experience both short and long term complications. Dietary intervention is the first therapeutic strategy. If good glycaemic control is not achieved, insulin therapy is recommended. There is no consensus on which nutritional approach should be used in GDM. In the last few years, there has been growing evidence of the benefits of a low glycaemic index (LGI) diet on diabetes and cardiovascular disease. The effect of a LGI diet on GDM incidence has been investigated as well. Several studies observed a lower incidence of GDM in LGI diet arms, without adverse maternal and fetal outcomes. The main positive effect of the LGI diet was the reduction of 2-h post-prandial glucose (PPG). Several studies have also evaluated the effect of the LGI diet in GDM treatment. Overall, the LGI diet might have beneficial effects on certain outcomes, such as 2-h PPG, fasting plasma glucose and lipid profile in patients with GDM. Indeed, most studies observed a significant reduction in insulin requirement. Overall, according to current evidence, the LGI nutritional approach is safe and it might therefore be considered in clinical care for GDM.

## 1. Introduction

Gestational diabetes mellitus (GDM) is defined as “diabetes diagnosed in the second or third trimester of pregnancy that was not clearly overt diabetes prior to gestation” [1]. Contextually with the dramatic spread of obesity and type 2 diabetes (T2D), the prevalence of GDM has significantly increased over the last few years. The prevalence of this condition depends largely on ethnicity and it is also related to the criteria employed for diagnosis [2].

Physiologically, during pregnancy insulin sensitivity progressively decreases until the second trimester, declining by 50%–60% compared with pre-pregnancy values [3]. Insulin sensitivity is influenced by the increase in adipose tissue, and by the release of hormones, such as estrogens and progesterone, or placental factors, such as human placental lactogen (hPL) [3]. In particular, hPL has a lipolytic effect, leading to the rise in circulating fatty acids. Consequently, maternal metabolism is shifted towards a greater use of lipids rather than glucose as an energy source, which is necessary to give the fetus an adequate glucose reserve [3]. Insulin resistance develops as a consequence of high maternal circulating fatty acids [3]. Contextually, insulin secretion is implemented to balance peripheral insulin resistance. However, if the increase in pancreatic β-cell secretion is not able to compensate insulin resistance, diabetes occurs [4]. Moreover, physiological pregnancy is characterized by low grade inflammation, which is exacerbated in GDM. Thus, insulin resistance might develop from this pro-inflammatory state [5].

The main risk factors for GDM are pre-pregnancy body mass index (BMI) in the range of overweight or obesity, high maternal age, and first degree family history of T2D [6,7].

GDM may lead to several adverse pregnancy outcomes. Indeed, mothers affected by GDM have a high prevalence of gestational hypertension and preeclampsia. The incidence of caesarean section in GDM patients is considerably higher than in normal pregnancies, as well as the occurrence of obstetrical complications, such as preterm delivery, dystocia, acute respiratory distress syndrome, neonatal jaundice, and neonatal hypoglycemia [8,9]. Besides short-term complications, women with previous GDM are at increased risk for GDM recurrence, T2D, cardiovascular disease (CVD), and hypertension. Furthermore, it has been observed that even the offspring of mothers with GDM has increased risk of obesity, insulin resistance, and T2D later in life [10].

As for diagnosis, a single-step approach has been proposed by the International Association of the Diabetes and Pregnancy Study Groups (IADPSG), which consists of performing an oral glucose tolerance test (OGTT) with 75-g of glucose and assessing fasting, 1 h and 2 h glycaemia. A single value higher than the cut-off value at any time of the OGTT is sufficient to establish the diagnosis [11]. The IADPSG criteria have been developed after the results of the Hyperglycemia and Adverse Pregnancy Outcome (HAPO) study, a multinational prospective study of 25,000 pregnant women, which observed that high glucose levels were significantly associated with increased risk of maternal and fetal complications [12].

The treatment of GDM is aimed to achieve normal levels of fasting plasma glucose (FPG) and post-prandial glycaemia (PPG) in order to prevent complications. Indeed, high glucose levels, especially postprandial elevations, are linked to negative pregnancy outcomes [13]. The first therapeutic approach consists of dietary and lifestyle education. However, insulin therapy is mandatory unless good glycaemic control is achieved [14]. Medical nutritional therapy should provide adequate nutrients for normal fetal growth, but it should not induce maternal weight gain or loss [15]. Gestational weight gain is largely due to excessive energy intake and fuel requirements usually increase from 10 to 30 weeks of gestation [16]. Caloric restriction is not recommended during pregnancy, even in GDM, because it may have adverse effects on birth weight. Advice for energy intake, as well as for weight gain, should take into account pre-pregnancy BMI [16,17]. As yet, there is no consensus on which specific nutritional approach should be used in GDM, in terms of total energy intake and macronutrient distribution [18].

This paper provides a brief overview on medical nutritional therapy in GDM, with a specific focus on the low glycaemic index (LGI) diet approach. To date, although several studies and meta-analyses have been performed, their results are not univocal and the LGI diet is still a debated topic in GDM. The most recent findings on this issue have been discussed and compared. In addition, the main limits of the latest studies and meta-analyses have been indicated.

## 2. Carbohydrates, Glycaemic Index, and Glycaemic Load

Carbohydrates (CHOs) are an important source of energy, but they increase PPG more than other macronutrients [19]. Since a proportion higher than 55% of CHO in diet is associated with elevations in PPG [20], moderation of CHO intake is reasonable in GDM. However, restriction of CHO is not mandatory to reduce PPG. The decrease in the rate of CHO digestion and absorption is effective in preventing PPG abnormal elevations. Not only is blood glucose influenced by the total amount of CHOs, but it is also affected by the type of CHO. CHO polymer length influences digestion and absorption, and consequently might prevent the elevations of glycaemia after meals.

Glycaemic response (GR), glycaemic index (GI), and glycaemic load (GL) are measures of the effect of different foods on glycaemia [21]. When a food containing CHO is ingested, the PPG variation induced is the GR [21]. “Available CHO” is the amount of CHO that goes through digestion, absorption, and metabolism. The GR induced by a portion of food containing 50 g of available CHO is the GI, which represents a percentage of the GR induced by 50 g of the reference CHO (generally glucose or white wheat bread) [21,22]. For instance, rice and potatoes are considered high GI foods, as they induce a rapid increase in glycaemia, which subsequently drops sharply. On the contrary, fruits and dairy are considered LGI foods, since they contain CHOs which are digested slowly and induce lower PPG elevations [16,21,22]. High GI foods have a GI ≥70 on the glucose scale and contain CHOs which are digested, absorbed and metabolized sharply. Whereas LGI foods have a GI ≤55 and are digested, absorbed and metabolized slowly. Thus, GI is a standardized measure of GR, defined by a standardized amount of CHO and it is relative to a reference food. It can also be considered as an indicator of the quality of CHO. The GL is a measure of both the quality and the amount of CHOs. It is calculated by multiplying the GI by the quantity of available CHO in a given amount of food (“GL = GI x available CHO/given amount of food”) [21]. The glycaemic effect of whole diets, meals, and foods can be easily compared on the basis of their GL [21,23].

The GI was defined for the first time many years ago by Jenkins et al. [24]. The International Organization for Standardization has developed the reference method, which is still valid [21,25], and international tables indicating the GI of foods are available [26]. Besides the adoption of GI for single foods classification and comparison, the use of GI has spread to mixed meals and entire diets as well. Indeed, meal and dietary GI employed in clinical trials and in clinical setting are obtained by calculating the weighted mean of the GI of each food, considering the proportion of CHO contained in them [27].

In addition, the content of dietary fibers (DF), plant-based CHO that are not digestible, is another important indicator of the quality of diets. DF contained in fruits, vegetables, and legumes are soluble. Soluble DF slow digestion, reduce PPG, and cholesterol absorption. Insoluble DF are contained in nuts, wholegrain bread, and cereals. Cooked potato and rice contain resistant starch [16]. Insoluble DF and resistant starch have limited metabolic actions [23].

## 3. LGI Diets and Risk of GDM

In the last 30 years there has been growing evidence of the benefits of the LGI diet not only on weight reduction and body composition but also on diabetes, CVD, and cancer. LGI food produces lower PPG increase, prevents the excessive rise in post-prandial insulin, and it might also induce satiety. All these effects may contribute to weight loss [28].

Besides weight reduction, in epidemiological studies the LGI diet was associated with a lower risk of T2D, CVD, and cancer, mainly breast and colorectal neoplasms [29,30,31]. Furthermore, in clinical studies, the LGI diet had positive effects on glycaemic control in diabetes, lipid profile, and other CV risk factors [31,32,33,34]. The mechanisms behind these beneficial effects remain not completely understood, but they might include slow absorption of CHOs and improvement even in novel cardiovascular risk factors, such as chronic inflammation.

A growing amount of research has focused on the effects of the LGI diet on the incidence of GDM as well. The ROLO study (“randomized control trial of low glycaemic index diet versus no dietary intervention to prevent recurrence of fetal macrosomia”) enrolled 800 women with previous macrosomia (infant >4 kg). In this study, women in the LGI diet group experienced significantly lower weight increase during pregnancy than women in the control group. Although the incidence of large for gestational age (LGA) infants was not influenced by the intervention diet, the incidence of glucose intolerance was lower in the LGI arm. Indeed, a lower percentage of mothers had fasting glycaemia ≥92 mg/dL or 1-h glucose at OGTT ≥140 mg/dL [35]. Later, the same authors performed a secondary analysis of 621 participants. A lower increase in circulating levels of insulin was observed from the beginning of pregnancy to 28 weeks of gestation, in women in the intervention group, although no differences were found between the two groups in maternal and fetal adipokines and inflammation markers (tumor necrosis factor α, interleukin 6, leptin) [36]. A further analysis of 542 mothers and babies previously involved in the ROLO study indicated a positive association between neonatal central adiposity and the LGI group [37].

In the GI Baby 3 Randomized Controlled Trial (RCT), Markovic et al. enrolled 139 women at high risk for GDM, between 14 and 20 weeks of pregnancy. The treatment arms were either an LGI diet with a target GI around 50 (72 women), or a conventional moderate-GI high DF diet, with a target GI around 60 (67 women). At 36 weeks, no differences in glycosylated hemoglobin (HbA1c) as well as in lipids, birth weight, percentage of fat of infants, and incidence of GDM were observed. In a subsequent prospective study, including a selection of 59 mothers and infant pairs (30 from the LGI group and 29 from the high-fiber group), infants had lower birth weight and length in the LGI group than in the high-fiber group. However, growth curves and fat mass from birth to 1 year of age were not significantly different. Notably, in the LGI group, babies had a significantly lower aortic intima-media thickness than in the high-fiber group. Even though subjects involved in the follow-up study were different from those initially assigned to the study, and although the follow-up study was under-powered in detecting differences in fat mass, these results suggest that GI might have an effect on the offspring vascular health [38].

A meta-analysis of 11 randomized controlled trials (1985 women) showed a significant reduction in FPG, 2-h PPG, and in the percentage of LGA in the LGI diet arm than in the control arm. Birth weight and weight increase during pregnancy were lower in the LGI arm, but not significantly, and the heterogeneity was high in gestational weight gain and birth weight analyses, probably due to different methods of intervention and control treatment, characteristics of participants, criteria for diagnosis of GDM. However, these data show that the LGI diet may improve several maternal metabolic outcomes, without negative effects on the infant [39].

Interestingly, total CHO intake in diet seems to be less important than CHO quality (DF content, GI, GL) in prevention of GDM. The Australian Longitudinal Study on Women’s Health aimed to investigate the relationship between the amount and the quality of pre-pregnancy CHOs in diet and the risk of developing GDM [40]. A total of 3607 women (25–30 years) were enrolled and followed up for a 10-year period. The low CHO diet score referred to high fat, high protein, and low CHO relative content. Paradoxically, this score and the risk of GDM were found to be positively correlated. However, the risk of GDM was reduced by 33% in women with the highest DF intake. The amount of fruits was negatively associated with GDM as well, whilst a high cereal content was a risk factor for GDM. Thus, it is crucial to take into account not only the quantity but also the source of CHO in diet, in terms of fiber content, GI, and GL.

Overall, it seems that clinical phenotypes with increased insulin resistance and therefore with high post-prandial insulin, higher BMI, or waist circumference, especially in the presence of diabetes, might benefit the most from the LGI diet. Several studies have indicated a lower incidence of GDM in pregnant women in LGI diet arms, without adverse maternal and fetal outcomes. The main positive effect of the LGI diet was found to be the reduction of 2-h PPG. Nevertheless, considering the high heterogeneity between studies in population characteristics, outcomes, intervention, and control treatment, and the relative limited number of studies considered in meta-analyses, further research should confirm these findings. Finally, the exact effect of DF content in the LGI diet in preventing GDM has not been elucidated so far.

## 4. LGI Diet and Pregnancy Outcomes in GDM

The efficacy of the LGI diet in GDM has been evaluated in several RCT and meta-analyses. Moses et al. performed an RCT, involving 63 GDM women. The treatment arms were either an LGI diet (31) or a standard high-fiber diet, with higher GI (32), and patients were followed from 28–32 weeks of pregnancy until delivery. A significantly lower percentage of patients required insulin treatment in the LGI diet arm than in the high-GI diet arm (29% vs. 59% respectively, *p* = 0.023). However, among the women needing insulin, a proportion of 47% avoided it by shifting to an LGI intervention. Obstetric and fetal outcomes, such as type of delivery, birth weight, length, cranial circumference, and Apgar index did not differ between the two groups. Notably, fiber intake, which may act as a confounding factor in evaluating the potential advantages of the LGI diet, was similar in both groups [41].

Hu et al. performed an RCT, randomizing 140 women with GDM to either the LGI diet (66) or normal diabetic control diet (74). In the LGI diet group rice was replaced by an LGI food in the principal meals. Remarkably, PPG levels were significantly lower in the LGI group than in the control group, although the two arms had similar total energy and CHO content [42].

Similarly, the LGI diet induced a decrease in FPG and 2-h PPG in an RCT involving 95 Chinese patients with GDM, assigned either to general dietary intervention or intensive LGL intervention, from 24–26 weeks of pregnancy until delivery. Total cholesterol, triglycerides, and HDL-cholesterol significantly improved in the LGI arm. No significant differences in other outcomes emerged between the two groups. However, since the DF intake was significantly greater in the intervention arm, the effects of the LGI and the high DF could not be differentiated [43].

In another RCT, 99 women with GDM were randomized to either an LGI diet (50) or a high fiber moderate GI diet (49) and followed up from 20–32 weeks of pregnancy until delivery. In contrast to the results of other studies, there were no significant differences in insulin treatment requirement or other maternal and fetal outcomes [44]. A subgroup of 58 pregnant women assigned either to the LGI diet (33) or to the high-fiber diet (25) was followed up further. Three months after delivery there were no significant differences in glucose levels after a 75-g OGTT and in blood lipids [45]. It should be noted that the latter results might be explained by a very modest difference in GI in diets between the two arms (target GI: Around 50 in the LGI arm and around 60 in high-fiber moderate-GI arm).

A meta-analysis of RCTs of dietary intervention in GDM showed that in the LGI group (4 RCT, 257 patients diet) the use of insulin was less frequent and the birth weight was lower than in the control group [46]. The energy intake did not differ between the groups and the mean value of GI score in the LGI dietary intervention was 48.9, whereas in the control group was 53.5. Remarkably, the LGI diet did not increase the number of small for gestational age (SGA) infants. As regarding macrosomia, no significant differences were observed, probably due to the relatively low incidence of this condition both in the intervention and in the control groups.

In another meta-analysis of 5 RCTs (302 patients with GDM) [47] the risk of insulin usage was reduced by 33% in the LGI diet, although not significantly. Since fiber content was considerably higher in LGI diets than in the control diets in two of the selected studies, in subgroup analyses the authors investigated the effect of this confounder on the specific outcome insulin usage. Considering the 3 studies in which the fiber intake was similar between the two groups, the risk of insulin requirement was significantly lower in LGI diets (−31%, *p* = 0.01). However, the increase in the content of DF in the LGI diet did not lead to a further reduction in the risk of insulin requirement. Besides this, the risk of macrosomia was significantly lower in the LGI groups than in the control groups and the risk of SGA was not statistically different between the two groups. Interestingly, the addition of DF to LGI diets further reduced the risk of macrosomia, compared with LGI diets without added fiber.

Recently, in a meta-analysis of 6 RCTs, including 532 patients with GDM (270 treated with the LGI diet vs. 262 treated with control-diet), the LGI diet lead to a significant reduction in 2 h PPG, without any influence on FPG, HbA1c, insulin requirement, birth weight, and macrosomia [48].

It is known that previous GDM increases the risk of T2D. In an RCT, 77 Asian women with previous GDM were assigned to either a standard healthy diet, which consisted of receiving advice on energy restriction, fat and simple sugar reduction, fiber content increase, or an LGI diet, with the addition of instructions for lowering the GI. After 6 months, body weight, BMI, waist-to-hip ratio, and 2 h PPG significantly reduced only in the LGI group. Notably, although caloric intake was similar between groups, fiber content was higher in the LGI diet group than in the control group (17 ± 4 vs. 13 ± 4 g, *p* < 0.001) [49]. The same authors investigated the effects of LGI by grouping patients according to fasting insulin levels at baseline. Considering the subjects with low levels of insulin, no significant differences in outcomes between the diet groups were observed. After 12 months, FPG and triglycerides reduced in normal or high insulin subjects in the LGI group, whereas in the conventional healthy dietary recommendation group an increase was observed [50]. These findings might indicate that clinical phenotypes with increased insulin resistance could take more advantage from LGI diets.

Several reasons may explain the main differences in the results between the above-mentioned studies. Firstly, different diagnostic criteria for GDM have been adopted. The variation in patient characteristics (age, ethnicity, weeks of gestation, BMI) and in the methods of intervention/control diets should be taken into consideration. Moreover, different or not standardized outcomes were evaluated. Furthermore, most studies were limited by the lack of assessment of adherence to diet. The relatively small number of some studies included in the meta-analyses (*n* < 100) is another important limitation. A further explanation for the contrasting findings between studies is the different amount and type of DF intake in treatment arms, which may act as a confounder.

## 5. Conclusions

Overall, a considerable body of research suggests that the LGI diet might have positive effects on certain metabolic outcomes, such as 2 h PPG, FPG, and likely, lipid profile in patients with GDM. Indeed, most clinical studies observed a significant reduction in insulin requirement. By contrast, the LGI diet has not shown to have a marked influence on other obstetric maternal or fetal outcomes (body weight gain, birth weight, proportion of LGA, and macrosomia) so far. Interestingly, current evidence suggests that an LGI nutritional approach is reasonably safe in GDM, since it was not associated to adverse fetal-maternal outcomes in clinical trials. In clinical care, giving LGI advice in pregnancy complicated by GDM might therefore be considered, especially in women with uncontrolled PPG or FPG, before starting insulin treatment. As yet, evidence about clear reduction in GDM incidence with the LGI diet is still insufficient. Similarly, further studies should focus on long term complications of GDM as well, such as the incidence of T2D and CVD in mothers and infants.
